# Effect of rituximab on long-term damage acquisition in patients with systemic lupus erythematosus

**DOI:** 10.1093/rheumatology/keaf248

**Published:** 2025-05-15

**Authors:** Amanda da Silva Brito, Sofia Miranda, Teresa Moitinho de Almeida, David A Isenberg

**Affiliations:** Serviço de Medicina Interna, Instituto de Medicina Integral Professor Fernando Figueira, Recife, Pernambuco, Brazil; Serviço de Medicina Interna, Hospital do Divino Espírito Santo de Ponta Delgada, São Miguel, Portugal; Serviço de Medicina Interna, Hospital Egas Moniz, Centro Hospitalar de Lisboa Ocidental, Lisboa, Portugal; Division of Medicine, Department of Ageing, Rheumatology and Regenerative Medicine, University College London, London, UK

**Keywords:** B-cell depleting agents, rituximab, systemic lupus erythematosus (SLE), lupus nephritis, damage index, cyclophosphamide, immunosuppressive agents, disease progression

## Abstract

**Objectives:**

B-cell depletion therapy has been used for over two decades to treat SLE, but there is a lack of studies reporting its impact on damage progression. This study aims to assess the effectiveness of rituximab in slowing damage acquisition.

**Methods:**

We selected 380 patients 190 treated with rituximab and 190 controls, based on matched sex and age of onset, with standard immunosuppressive therapies—to compare the damage they developed, assessed by the SLICC/ACR Damage Index (DI). A secondary analysis of 111 patients was conducted to evaluate DI progression.

**Results:**

The majority of patients were female (94.1%) and Caucasian (45.4%). Severe disease manifestations and higher titres of anti-dsDNA antibodies (86 U/ml *vs* 62 U/ml; *P =* 0.012) were seen in the rituximab group, in which SLICC/ACR DI was also higher (1.3 *vs* 0.9; *P =* 0.02). In the secondary analysis the SLICC/ACR DI mean had no statistical difference between the two groups (0.4 *vs* 0.6; *P =* 0.33), but we identified a statistical significance between the two groups regarding their DI progression (58.2% in the control group *vs* 44.2% in the rituximab).

**Conclusion:**

As an effective B-cell depleting therapy, rituximab is a valid therapeutic option for SLE patients, especially in those with refractory or life-threatening manifestations. While patients treated with rituximab initially had higher damage, their rate of damage progression was slower compared with those receiving standard therapies.

Rheumatology key messages

- Rituximab effectively manages refractory SLE, especially LN and neuropsychiatric lupus.
- Long-term rituximab use does not reduce damage significantly compared with standard immunosuppressive therapies in SLE.
- Rituximab enables significant CS sparing in SLE, improving long-term disease management and outcomes.

## Introduction

B-cell depletion has been used to treat patients with SLE for over 20 years [1]. In spite of the failure of two large clinical trials (one renal [2] and one non-renal [3]) to meet the primary endpoints, greater success has been reported using rituximab, either alone or in combination with CYC, in numerous cohort and case studies [4–9]. It is furthermore recommended for the treatment of LN by both the ACR [10] and the EULAR [11]. Its use in SLE is comparable to its widespread use, following successfully conducted trials in rheumatoid arthritis [12], vasculitis [13] and idiopathic membranous nephropathy [14]. In SLE patients, the main focus of interest has been on the capacity of rituximab to reduce activity. Much less attention has been paid as to whether in the longer term, it has any effect on reducing the acquisition of damage. Damage reduction is important in SLE. As has been shown, early acquisition of damage within a year [15] and within a decade [16] are both indicators of increased risk of mortality. We have sought to determine whether rituximab might be at least as good as conventional immunosuppression in restricting damage acquisition. We have used our large cohort of patients (over 850 patients treated between 1978 and 2023) to try and answer this question. In the past two decades, we have treated just over 200 patients with rituximab, mostly to those who were still active in spite of being given steroids, HCQ and standard immunosuppressives such as AZA, CYC and MMF. We now present a comparison of those patients treated with rituximab in whom we had adequate follow-up (1 year minimum) and those given more standard immunosuppressive therapies.

## Methods

Between 1978 and 2023, we treated a cohort of 850 patients diagnosed with SLE based on the 1997 ACR revised criteria and the 2019 ACR/EULAR criteria. We lack studies comparing damage in SLE patients who used rituximab with those who did not. However, estimating twice the risk for rituximab (due to refractory disease) and aiming for 80% power with a 95% two-sided confidence level, the total number of patients required was 240, with 118 patients needed per group. From our cohort, 380 adult patients treated at the SLE Clinic of University College Hospital were carefully selected for retrospective analysis, using our almost 200 patients who had used rituximab as our standard group. These patients were categorized into two treatment groups: 190 patients who received rituximab and 190 who received standard immunosuppressive therapies, based on sex and age of onset matching. Furthermore, a secondary analysis was performed on a subgroup of 111 patients. Here two groups were compared, one with rituximab treated patients; each of whom was more meticulously matched with two counterparts based on sex, age at onset (the rituximab treated and control patients had to be within 5 years of each other), type of SLE (renal or non-renal), activity and ethnicity. This matching process enabled a detailed comparative analysis of disease progression and the damage index between the rituximab group and the control group. This approach was particularly critical as it accounted for the likelihood of rituximab being administered to patients with more refractory forms of the disease. Comprehensive medical record reviews were conducted to analyse demographics, clinical presentations, serological markers, prior and concurrent treatments, rituximab treatment protocols, and adverse events—including serious infections. Serious infections were considered those which resulted in the hospitalization of the patient. Our primary outcome on the first analysis was difference between the SLICC/ACR Damage Index (DI) after the variable follow-up. The follow-up period was defined as the time from the initial consultation post-SLE diagnosis to the last routine visit, with a minimum duration of 1 year. The study was an audit, not requiring formal hospital ethics approval due to the observational retrospective nature of the study using de-identified data—no individualized or identifiable data are presented in this study. Therefore, informed consent was not required. Statistical evaluations were performed on both categorical and continuous variables. Categorical variables were analysed using relative and absolute proportions, while continuous variables were examined using medians, means and interquartile ranges to assess data variability. Group comparisons were made using Pearson’s χ^2^ test or Fisher’s exact test as appropriate. For normally distributed quantitative data, the Student’s *t*-test was utilized, whereas the Mann–Whitney test was employed for nonparametric data. For the secondary analysis, we used a generalized estimating equation to evaluate the damage index variations through both groups and through the follow-up. A *P*-value of <0.05 was considered statistically significant. All analyses were conducted using the SPSS software, version 21, for Windows.

## Results

The majority of patients (*n* = 190) were female, 94.1% of the participants, with a median age of 47 years. Patients in the control group (*n* = 190) were significantly older than those in the rituximab group (median ages 50.1 *vs* 45.9 years, respectively; *P =* 0.002). The median age at which patients first showed symptoms of the disease was 25 years, with no significant difference between groups (26 years in the control group *vs* 25 years in the rituximab group; *P =* 0.068). In terms of ethnicity, 51.8% *vs* 39% of the patients were Caucasian in the control group *vs* the rituximab group, respectively, making it the most represented group, followed by Asian (18.5% in the control *vs* 23.1% in the rituximab group). These demographic characteristics are detailed in Table 1.

**Table 1.** Demographic and baseline disease characteristics of study population

Primary analysis				
	Total (*N* = 390)	Group		*P*-value
		Control (*n* = 195)	Rituximab (*n* = 195)	
Sex, *n* (%)				
Female	367 (94.1)	187 (95.9)	180 (92.3)	0.132
Male	23 (5.9)	8 (4.1)	75 (7.7)	
Age (years)				
Median (min–max)	47 (19–86)	50 (19–86)	44 (21–86)	0.002
Age of onset (years)				
Median (min–max)	25 (4–70)	26 (8–58)	25 (4–70)	0.068
Ethnicity, *n* (%)				
Asian	81 (20.8)	36 (18.5)	45 (23.1)	
Black	7 (1.8)	5 (2.6)	2 (1.0)	
Black African	32 (8.2)	12 (6.2)	20 (10.3)	
Black Caribbean	57 (14.6)	22 (11.3)	35 (17.9)	
Caucasian	177 (45.4)	101 (51.8)	76 (39.0)	0.027
Mixed	13 (3.3)	4 (2.1)	9 (4.6)	
Other	23 (5.9)	15 (7.7)	8 (4.1)	
Time of follow-up (years)				
Median (min–max)	14 (1–44)	14 (1–44)	15 (1–31)	0.398
Years of disease				
Median (min–max)	21 (1–55)	22 (1–55)	20 (2–47)	0.045^∗^


Secondary analysis				
	Total (*N* = 111)	Control (*N* = 74)	Rituximab (*N* = 37)	*P*-value
Sex, *n* (%)				
Female	105 (94.6)	70 (94.6)	35 (94.6)	1.0
Male	6 (5.4)	4 (5.4)	2 (5.4)	
Age (years)				
Mean (s.d.)	51.8 (12.4)	52.7 (12.3)	49.9 (12.7)	0.272
Ethnicity, *n* (%)				
Asian	21 (18.9)	12 (16.2)	9 (24.3)	0.784
Black	21 (18.9)	15 (20.3)	6 (16.2)	
Caucasian	65 (58.6)	44 (59.4)	21 (56.8)	
Other	4 (3.6)	3 (4.1)	1 (2.7)	

∗ *P*-value < 0.05.

In terms of clinical features, the control group exhibited a slightly longer duration of disease compared with the rituximab group (22 *vs* 20 years, respectively; *P =* 0.045). Severe disease manifestations, which are crucial in determining adequate therapeutic approaches, including prevalence of LN (56.9% *vs* 38.1%; *P* < 0.001) and neuropsychiatric lupus (22.6% *vs* 12.8%; *P =* 0.012), were higher in the rituximab group. A higher number of patients in the rituximab group developed renal failure (19% *vs* 11.3%; *P =* 0.034); however, this did not result in a significant increase in the transplantation rates (5.6% *vs* 2.6%; *P =* 0.126). No difference in mortality was seen between groups (10.8% *vs* 10.8%; *P* > 0.99). Constitutional symptoms (71.8% *vs* 61.5%; *P =* 0.032), cardiopulmonary disease (39.4% *vs* 26.2%; *P =* 0.005), musculoskeletal symptoms (93.8% *vs* 86.2%; *P =* 0.011), haematologic disease (57.4% *vs* 47.2%; *P =* 0.043) and vasculitis (21.5% *vs* 9.2%; *P =* 0.001) were also more prevalent among those who used rituximab, but gastrointestinal, mucocutaneous and ophthalmological manifestations did not differ between groups. When we analysed the serological profile of the groups, there were no statistically significant differences in complement consumption (34.4% *vs* 25.6%; *P =* 0.06); however, the rituximab group exhibited significantly higher titers of anti-dsDNA antibodies (62 *vs* 86 U/ml; *P =* 0.012). The clinical and serological features are detailed in Table 2.

**Table 2.** Clinical and serological features of both groups

	Total (*N* = 390), *n* (%)	Group		*P*-value^∗^
		Control (*n* = 195), *n* (%)	Rituximab (*n* = 195), *n* (%)	
LN	185 (47.6)	74 (38.1)	111 (56.9)	<0.001^*^
Neuropsychiatric lupus	69 (17.7)	25 (12.8)	44 (22.6)	0.012^*^
Renal failure	59 (15.1)	22 (11.3)	37 (19.0)	0.034^*^
Renal transplant	16 (4.1)	5 (2.6)	11 (5.6)	0.126
Constitutional symptoms	260 (66.7)	120 (61.5)	140 (71.8)	0.032^*^
Cardiopulmonary disease	128 (32.8)	51 (26.2)	77 (39.4)	0.005^*^
Musculoskeletal	351 (90.0)	168 (86.2)	183 (93.8)	0.011^*^
Hematologic	204 (52.3)	92 (47.2)	112 (57.4)	0.043^*^
Vasculitis	60 (15.4)	18 (9.2)	42 (21.5)	0.001^*^
Gastrointestinal	10 (2.6)	2 (1.0)	8 (4.1)	0.105
Cutaneous SLE	324 (83.1)	157 (80.5)	167 (85.6)	0.177
Ophtalmological	5 (1.3)	0 (0)	5 (2.6)	0.061
Reduced complement	117 (30.0)	50 (25.6)	67 (34.4)	0.060
Positive anti-dsDNA	148 (37.9)	62 (31.8)	86 (44.1)	0.012^*^
Mortality	42 (10.8)	21 (10.8)	21 (10.8)	>0.999

∗ *P*-value < 0.05.

For the rituximab group, SLICC/ACR DI mean was higher at the beginning of the follow-up compared with the control group (1.3 *vs* 0.9; *P =* 0.023). In addition, the proportion of participants with SLICC/ACR DI of zero was lower in the rituximab group (40%) compared with the control group (53.3%), and this difference is statistically significant with a *P*-value of 0.008. Regarding our secondary outcomes, there were no significant difference between the incidence of cancer (7.7% in the rituximab group *vs* 11.3% in the control group; *P =* 0.226) or serious infections (19.5% in the rituximab group *vs* 13.3% in the control group; *P =* 0.101). Patients received various combinations of the available recommended immunosuppressive therapies according to disease manifestations and severity. Our data showed no significant difference in HCQ use between the two groups (64.1% in the rituximab group *vs* 62.1% in the control group; *P =* 0.675). Use of MMF was higher in the rituximab group (32.3% *vs* 9.7%; *P* < 0.001), as was the use of tacrolimus (4.6% in the rituximab group *vs* 0.5% in the control group; *P* < 0.01). No difference was seen in other standard immunosuppressive drugs such as AZA (16.9% in the control *vs* 13.4% in the rituximab group; *P =* 0.33), MTX (5.1% in controls *vs* 4.1% in rituximab patients; *P =* 0.629) or ciclosporin (2.6% in controls *vs* 0.5% in rituximab patients; *P =* 0.215). Regarding the use of biologic drugs, there was no statistical difference between the groups, as shown in Table 3. The few patients who had post-rituximab immunosuppression using obinutuzumab, belimumab, tocilizumab, Janus kinase inhibitors or TNF-α blockers were under occasional special circumstances, mostly due to an overlap syndrome (e.g. RA). The use of CS at the end of follow-up was also significantly higher in the rituximab group (72.3%) compared with the control group (50.8%) (*P* < 0.001); the average doses of steroids at the end of follow-up were similar between the groups and did not show a statistically significant difference.

**Table 3.** Damage index comparison and immunosuppression

Variable	Total (*N* = 390)	Group		*P*-value^∗^
		Control (*n* = 195)	Rituximab (*n* = 195)	
Primary analysis				
Years of disease				
Mean (s.d.)	22.8 (8.8)	23.5 (8.2)	21.5 (9.8)	
Median (min–max)	22 (4–47)	22 (4–42)	19 (6–47)	0.255
Initial damage index				
Mean (s.d.)	0.5 (0.9)	0.3 (0.7)	0.9 (1.3)	
Median (min–max)	0 (0–5)	0 (0–4)	1 (0–5)	0.002
Final damage index				
Mean (s.d.)	1.1 (1.5)	0.9 (1.4)	1.3 (1.5)	
Median (min–max)	1 (0–11)	0 (0–6)	1 (0–11)	0.023
Damage index, *n* (%)				
Zero	182 (46.7)	104 (53.3)	78 (40)	
≥1	208 (53.3)	91 (46.7)	117 (60)	0.008
HCQ	246 (63.1)	121 (62.1)	125 (64.1)	0.675
MMF	82 (21.0)	19 (9.7)	63 (32.3)	<0.001
MTX	18 (4.6)	10 (5.1)	8 (4.1)	0.629
Belimumab	5 (1.3)	1 (0.5)	4 (2.1)	0.372
Obinutuzimab	2 (0.5)	0 (0)	2 (1.0)	0.499
Tocilizumab	1 (0.3)	0 (0)	1 (0.5)	>0.999
JAK inhibitor	1 (0.3)	0 (0)	1 (0.5)	>0.999
TNF inhibitor	3 (0.8)	1 (0.5)	2 (1.0)	>0.999
Ciclosporin	6 (1.5)	5 (2.6)	1 (0.5)	0.215
CS	240 (61.5)	99 (50.8)	141 (72.3)	<0.001
Dose of CS				
Mean (s.d.)	6.0 (3,5)	5.7 (3.1)	6.2 (3.7)	
Median (min–max)	5 (1–30)	5 (1–20)	5 (1–30)	0.101
Cancer, *n* (%)	37 (9.5)	22 (11.3)	15 (7.7)	0.226
Serious infections, *n* (%)	64 (16.4)	26 (13.3)	38 (19.5)	0.101
Secondary analysis				
Damage index				
Initial mean (s.d.)	0.5 (0.9)	0.3 (0.7)	0.9 (1.3)	0.33
End mean (s.d.)	0.9 (1.4)	0.7 (1.2)	1.5 (1.6)	
Progression of damage index (%)		58.2	44.2	
Serious infection, *n* (%)	9 (8.3)	2 (2.7)	7 (19.4)	0.006^*^
Cancer, *n* (%)	10 (9.1)	7 (9.6)	3 (8.1)	0.999
Corticosteroids, *n* (%)	60 (54.1)	31 (41.9)	29 (78.4)	<0.001^*^
Dose of CS				
Median (min–max)	5 (1–15)	5 (1–15)	5 (3–13)	

JAK: Janus kinase.

∗ *P*-value < 0.05.

To assess the evolution of organ damage over time, we conducted a subgroup analysis in which patients were stratified into three diagnostic periods (1978–2000, 2000–2010 and 2010–2023) based on their year of initial diagnosis. This division accounts for differences in disease duration. Our aim was to determine whether earlier patients, particularly those treated before 2000—who initially had no access to MMF or rituximab—had a higher damage index. However, no statistically significant differences were found (*P* > 0.5) across all periods, as shown in Table 4. No significant subgroup differences were found between the damage index of patients with neuropsychiatric lupus treated with rituximab *vs* control (1.77 *vs* 1.48; *P =* 0.667) or the damage index between the ones with renal involvement treated with rituximab *vs* controls (1.13 *vs* 0.91; *P =* 0.33) as shown in Table 4.

**Table 4.** Subgroup analysis of damage index in SLE: analysis by time periods and disease severity

Damage Index (median)			
Period	Rituximab (*N* = 195)	Control (*N* = 195)	
	Mean of DI/total of patients	Mean of DI/total of patients	
1978-2000 (*N* = 109)	0.7 (*N* = 78)	0.38 (N = 31)	*P* > 0.5
2001–2010 (*N* = 169)	1.01 (*N* = 83)	1.03 (*N* = 86)	
2011-2023 (*N* = 110)	1.95 (*N* = 32)	1.17 (*N* = 78)	

DI (median)			
Neurolupus	Rituximab (*N* = 195)	Control (*N* = 195)	
Yes	1.77 (*N* = 44)	1.48 (*N* = 25)	*P =* 0.6667
No	1.14 (*N* = 151)	0.97 (*N* = 170)	

DI (median)			
Renal SLE	Rituximab	Control	
Yes	1.13 (*N* = 111)	0.91 (*N* = 74)	*P =* 0.33
No	1.40 (*N* = 84)	1.08 (*N* = 121)	

DI: Damage Index.

We conducted a secondary analysis using a smaller group of patients that were matched with two counterparts based on sex, age at onset, type of SLE (renal or non-renal), disease activity and ethnicity. We compared the variation of the SLICC/ACR DI from the first consultation until the end of follow-up to see whether rituximab slowed the progression of damage during our follow-up. The majority of the patients in this analysis remained female (94.6% in the control group *vs* 94.6% in the rituximab group), with a mean age of 52.7 years in the control group *vs* 49.9 years in the rituximab group. In both groups, most patients were Caucasian (59.4% *vs* 56.8% in the control and rituximab groups, respectively). This analysis showed that the mean SLICC/ACR DI of the control group at the beginning of follow-up was 0.3 compared with 0.9 in the rituximab group (*P =* 0.002). At the end of follow-up, this mean increased to 0.7 in the control group and to 1.5 in the rituximab group (*P =* 0.003), remaining higher among patients that underwent the use of rituximab. When we analysed the results from generalized estimating equations, we identified statistical significance between the two groups regarding the proportion of patients that progressed to a higher damage index. In the control group, 58.2% ended the follow-up of the patients with a higher SLICC/ACR DI, while in the rituximab group this change occurred in only 44.2% of patients. The mean variation of SLICC/ACR DI at the beginning and at the end of the follow-up showed no statistical difference between the two groups (0.4 *vs* 0.6; *P =* 0.33).

## Discussion

Our long-term analysis of 390 patients did not demonstrate a significant reduction in damage as captured by the SLICC/ACR DI in those treated with rituximab compared with patients receiving standard therapies. It is well established that as an effective B-cell depleting therapy, rituximab is a valid therapeutic option for SLE patients, especially in those with refractory or life-threatening manifestations [5, 6], notably LN [9] and neuropsychiatric lupus [10]. This patient profile is compatible with what was seen in the rituximab group of our cohort, reflecting the fact that the patients that underwent treatment with rituximab had more severe disease from the beginning of our follow-up, as it frequently was prescribed after failure of standard treatments, and therefore a higher SLICC/ACR DI. This result aligns with previous studies which demonstrated the benefits of rituximab in severe cases of SLE [17, 18].

Assuming the higher SLICC/ACR DI was a result of a more aggressive SLE phenotype, we conducted a secondary analysis to evaluate disease progression, presuming that rituximab might have an effect in slowing disease progression by reducing the variation in SLICC/ACR DI, regardless of initial disease severity. We observed no difference in the variation of the SLICC/ACR DI during the follow-up of our matched group, suggesting that rituximab could not slow damage progression. However, there was an absolute difference between the two groups when we compared the number of cumulative points in the damage index favouring rituximab. This observation highlights the need for further research to explore the role of rituximab in preventing long-term damage and identifying which patients might benefit the most from this therapy. Further analysis showed that, over time, a higher proportion of patients in the rituximab group required additional immunosuppressive therapies, such as MMF and tacrolimus, by the end of the follow-up period. This could imply that patients receiving rituximab had a more aggressive or refractory form of the disease, necessitating additional therapies to manage disease activity. The combination of rituximab with MMF has already been associated with a lower risk of disease flare in some studies [19] and the combination of rituximab with calcineurin inhibitors has been suggested as a promising approach for managing refractory LN [14, 20].

We found a relatively high rate of serious infections (19.5%) among patients treated with rituximab during follow-up, slightly higher than compared with previous data, such as the Spanish Registry (11%) [19, 21]. This rate of serious infections associated with rituximab underscores the importance of careful monitoring and management of side effects in patients receiving this treatment.

The issue of CS used in patients with SLE treated with rituximab remains contentious. Some studies [7] suggest that rituximab may enable a reduction in CS use, while others have not observed a significant difference [22]. In our study, despite the higher number of patients in the rituximab group requiring CS due to disease severity, we found no significant difference in the average doses of CS between the groups. Our average dose at the end of follow-up was 5 mg/day, which was lower compared with previous studies such as the French Cohort [7] (29.9 mg/day) and the EXPLORER study (45.9 mg/day) [3].

Furthermore, the study underscores the complexity of managing SLE, particularly in patients with severe or refractory forms of the disease. The decision to use rituximab must be balanced against potential risks, including increased infection rates and the possible need for additional immunosuppressive therapies. Clinicians should evaluate carefully individual patient characteristics, disease severity and response to previous treatments when considering rituximab as a therapeutic option. Developing strategies to identify patients at higher risk of complications and optimizing the use of rituximab in combination with other therapies could help improve outcomes and safety for SLE patients. In conclusion, while rituximab is a valuable tool in the management of severe SLE, our study suggests that it does not, overall, provide a significant advantage in reducing long-term damage compared with alternative therapies. However, our data suggest that it can be a helpful tool for maintaining patients on low doses of CS. This is encouraging as it has been shown [23] that a major cause of damage in SLE patients is the use of CS; thus anything which helps reduce the dose is most helpful. The high rate of serious infections observed in our cohort highlights the need for careful patient monitoring and management, especially in patients with multiple immunosuppressive drugs. Continued research is essential to optimize treatment strategies and improve outcomes for patients with SLE. By addressing these challenges, we can enhance our understanding of SLE management and work towards more effective and individualized therapeutic approaches.

## Data Availability

Summary statistics and deidentified data can be accessed upon appropriate request.
